# Clarifying values: an updated review

**DOI:** 10.1186/1472-6947-13-S2-S8

**Published:** 2013-11-29

**Authors:** Angela Fagerlin, Michael Pignone, Purva Abhyankar, Nananda Col, Deb Feldman-Stewart, Teresa Gavaruzzi, Jennifer Kryworuchko, Carrie A Levin, Arwen H Pieterse, Valerie Reyna, Anne Stiggelbout, Laura D Scherer, Celia Wills, Holly O Witteman

**Affiliations:** 1 Department of Internal Medicine and Center for Bioethics and Social Sciences in Medicine, University of Michigan and VA Ann Arbor Center for Clinical Management Research, 2800 Plymouth Road, Building 16, Rm. 455S, Ann Arbor, MI 48109-2800, USA; 2 Department of Medicine and Cecil Sheps Center for Health Services Research, CB# 7110, University of North Carolina, Chapel Hill, NC 27599, USA; 3 Nursing, Midwifery and Allied Health Professions Research Unit, University of Stirling, Iris Murdoch Building, Stirling, FK9 4LA, UK; 4 Departments of Family Medicine and Geriatric Medicine, College of Osteopathic Medicine and Center for Excellence in the Neurosciences, University of New England, Biddeford, ME 04005, USA; 5 Division of Cancer Care and Epidemiology, Cancer Research Institute, Department of Oncology, Queen’s University, Kingston, K7L 3N6, Canada; 6 Department of Developmental Psychology and Socialization, University of Padova (Italy), Via Venezia, 835131 Padova, Italy; 7 Leeds Institute of Health Sciences, Charles Thackrah Building, University of Leeds, 101 Clarendon Road, Leeds, LS2 9LJ; 8 University of Saskatchewan College of Nursing, St. Andrew’s College, 312, 1121 College Drive, Saskatoon, SK, S7N 0W3, Canada; 9 Informed Medical Decisions Foundation, 40 Court Street, Suite 300, Boston, MA 02108, USA; 10 Department of Medical Decision Making, Leiden University Medical Center, The Netherlands; 11 Center for Behavioral Economics and Decision Research and Cornell Magnetic Resonance Imaging Facility, Cornell University MVR B44, Ithaca, New York, 14853, USA; 12 Department of Medical Decision Making, Leiden University Medical Center, The Netherlands; 13 Department of Psychological Sciences, University of Missouri-Columbia, Columbia, MO, USA; 14 The Ohio State University College of Nursing, 384 Newton Hall, 1585 Neil Avenue, Columbus, OH 43210, USA; 15 Office of Education & Continuing Professional Development and Department of Family and Emergency Medicine, Université Laval, Pavillon Ferdinand-Vandry, bureau 2881-F, 1050 avenue de la Médecine, Quebec City, QC, G1V 0A6, Canada

## Abstract

**Background:**

Consensus guidelines have recommended that decision aids include a process for helping patients clarify their values. We sought to examine the theoretical and empirical evidence related to the use of values clarification methods in patient decision aids.

**Methods:**

Building on the International Patient Decision Aid Standards (IPDAS) Collaboration’s 2005 review of values clarification methods in decision aids, we convened a multi-disciplinary expert group to examine key definitions, decision-making process theories, and empirical evidence about the effects of values clarification methods in decision aids. To summarize the current state of theory and evidence about the role of values clarification methods in decision aids, we undertook a process of evidence review and summary.

**Results:**

Values clarification methods (VCMs) are best defined as methods to help patients think about the desirability of options or attributes of options within a specific decision context, in order to identify which option he/she prefers. Several decision making process theories were identified that can inform the design of values clarification methods, but no single “best” practice for how such methods should be constructed was determined. Our evidence review found that existing VCMs were used for a variety of different decisions, rarely referenced underlying theory for their design, but generally were well described in regard to their development process. Listing the pros and cons of a decision was the most common method used. The 13 trials that compared decision support with or without VCMs reached mixed results: some found that VCMs improved some decision-making processes, while others found no effect.

**Conclusions:**

Values clarification methods may improve decision-making processes and potentially more distal outcomes. However, the small number of evaluations of VCMs and, where evaluations exist, the heterogeneity in outcome measures makes it difficult to determine their overall effectiveness or the specific characteristics that increase effectiveness.

## Background

Consensus recommendations have indicated that decision aids should include some method to help patients^a^ consider how they value key aspects of the decision with which they are faced [1]. These recommendations are based on the belief that, by clarifying individuals’ values, the medical treatments that people actually receive will be more reflective of their personal preferences and treatment goals [2,3]. Whether such recommendations have strong theoretical and empirical justification remains controversial [4,5]. In fact, there is debate about whether patients’ participation in values clarification actually improves the quality of their decision making [2].

The purpose of this paper is to summarize the current state of theory and evidence with respect to the role of values clarification methods (VCMs) in patient decision aids. Through this overview, we hope to clarify when and how values clarification methods are used within decision aids, and to determine what further research needs to be done to improve the design and use of values clarification methods.

To better understand the state of the science pertaining to the inclusion of values clarification methods within decision aids, we undertook a process of evidence review and summary, described in greater detail below. Briefly, an international, multi-disciplinary committee of researchers in the field conducted a series of meetings over 18 months. Two sub-groups reviewed the relevant chapter in the International Patient Decision Aid Standards (IPDAS) Collaboration’s 2005 Background Document [6]; each sub-group was charged with one of two tasks. The theory and definitions sub-group was charged with developing an updated set of definitions and a summary of relevant theory that describes decision-making processes and their implications for values clarification [7]. The evidence sub-group was charged with conducting a systematic review of VCMs used and tested within decision aids [8]. Work from the two sub-committees was combined and edited to produce this report.

## An updated definition of values clarification methods

The relevant chapter in the IPDAS Collaboration’s 2005 Background Document uses the term “values clarification exercises”, and defines these as “[Exercises to] help patients to clarify and communicate the personal value of options, in order to improve the match between what is personally most desirable and which option is actually selected.” [6]

In this update, in order to improve clarity, we chose to set aside the term “*exercises*” and use the term “values clarification *methods*”. We then defined values clarification methods (VCMs) as strategies that are intended to help patients evaluate the desirability of options or attributes of options within a specific decision context, in order to identify which option he/she prefers.

Thus, our updated definition differs from the 2005 definition in two major ways. First, it focuses not only on the attributes of options (e.g., the probability of cure, the impact on bladder functioning), but also on the options as whole entities (e.g., the holistic comparison of surgery to radiotherapy), as well as on the decision context (e.g., the option that the doctor recommends, the option that my partner/children prefer). We use this broad definition because any of the above aspects of the situation—attributes, entities, context—may be relevant during the process of clarifying which option an individual prefers. Secondly, our updated definition does not include the communication of values to others, since this is considered to be a different aspect of the design of decision support interventions [2,3]).

Although values clarification methods can be either implicit and non-interactive (e.g., the patient thinks about what’s important to his decision) or explicit and interactive (e.g., the patient sets a rating scale for each attribute to reflect the importance of each to his decision) [6,9], this paper is focused on the more studied and better understood explicit values clarification methods.

## Theoretical rationale for evaluating patient decision aids on this quality dimension

### Original theoretical rationale

In the relevant chapter in the IPDAS Collaboration’s 2005 Background Document [6], different types of VCMs were described, including the mechanisms by which these methods may help patients to clarify their values. These mechanisms included:

1. Considering detailed information about the options and their outcomes, which helps to promote understanding of what it means to undergo the procedures involved and to face the physical, emotional, and social consequences;

2. Considering how others value features of options and whether the participant is similar to others (social matching); and

3. Rating or ranking features of options or trading off features of options, which may give insight into one’s personal values and/or the tradeoffs underlying the choice for one versus other options.

Note that these mechanisms did not refer to underlying theories.

### An updated theoretical rationale

We argue that VCMs are deemed to be helpful to patients because they provide assistance with particular decision processes. Decision-making process theories may provide useful guidance for us to gain better understanding about why or how values clarification methods affect decision processes. The theories presented in Additional file 1 Table S1a were selected because they specify particular decision processes, and hence provide the basis from which the subset of processes that VCMs may be able to assist were derived. These theories are described in greater detail in Additional file 2 Appendix 1. (Because some theories, such as Expected Utility Theory (EUT) and theories of behavioral change (e.g., Transtheoretical Model), do not specify particular decision processes, they cannot guide identification of the processes that VCMs might be able to help.)

#### Relevant theory-informed processes underlying values clarification

Several decision-making processes are relevant to values clarification. A specific VCM need not aim to address all decision-making processes, but should aim to facilitate, explicitly or implicitly, at least one or more of the following decision-making processes:

1. Identifying options, which can include either the narrowing down of options, or the generation of options that were not offered at the outset.

2. Identifying attributes of the situation and/or the options which ultimately affect the patient’s preference in a specific decision context.

3. Reasoning about options or attributes of options.

4. Integrating attributes of options using either compensatory or both compensatory and non-compensatory decision rules. (A compensatory decision rule allows the impact of an option’s attributes to be averaged so that a poor score on one attribute can be offset by a high score on a second attribute. A non-compensatory decision rule is a strategy that avoids tradeoffs; for example, a strategy in which the decision maker decides on a threshold that a particular attribute must meet for options to remain under consideration. Each option is evaluated on its own without consideration of other options)

5. Making holistic comparisons.

6. Helping decision makers retrieve relevant values from long-term memory.

Additional file 1 Table S1b provides examples of these decision-making processes, in the context of decision making about prostate cancer treatment.

## Empirical evidence: rationale, recent studies, evaluation

Below, the first sub-section presents a basic empirical rationale for evaluating the quality of a patient decision aid in terms of the method(s) it uses to foster values clarification. Then we outline the results of our updated review of the empirical evidence about the inclusion of VCMs in patient decision aids in two further sub-sections: the recent studies that have included VCMs in patient decision aids; and the evaluation of VCMs.

### An empirical rationale for evaluating patient decision aids on this quality dimension

Considerable evidence suggests that individuals facing new and complex decisions often do not have stable or clear preferences [10,11]. For example, Feldman-Stewart et al. showed that almost all early-stage prostate cancer patients who made use of a decision aid made changes from pre-test to post-test (i.e., within several hours) to the attributes that they identified as affecting their decision [12]. Importantly, these were patients who had already talked to their urologist and their radiation oncologist, and may have become clearer about what was important to them during those discussions. Based on the empirical evidence suggesting that patients may have unclear or unstable preferences, we argue that it continues to be important, when evaluating the quality of patient decision aids, to take into consideration the methods (if any) that are used to foster values clarification.

In the relevant chapter in the IPDAS Collaboration’s 2005 Background Document [6], the authors found 19 studies that employed VCMs [6]. They noted that most (72%) offered examples of how the values of other patients who had to make this decision led them to make different choices, and almost half (42%) incorporated some explicit means of measuring one’s values (e.g., rating, trade-offs, balance scales). However, few studies had examined the specific effects of including a VCM in a patient decision aid. Given the paucity of early evidence about the effects of VCMs in patient decision aids, we argue that our updated review of more recent relevant studies is timely.

### Recent studies that have included VCMs within decision aids

Witteman et al. conducted a rigorous systematic review of VCMs, and identified 61 VCMs that were used within a decision aid through June 2011 [8]. In their review, Witteman and colleagues examined a large number of the characteristics of these VCMs and of the decision aids in which they were embedded. We present a sub-set of these data here. Our goal was to describe the state of the science of studies that include VCMs. Thus, the factors we ultimately decided to highlight were based on the following principles: 1) the characteristics of the studies in which the VCMs were presented (e.g., the decision context), 2) the characteristics influencing how a particular VCM was originally designed (e.g., the development process), and 3) the characteristics of the VCMs themselves (e.g., the type of VCM utilized). Our review is therefore limited to “higher level” characteristics of the VCMs and the studies in which they were included. Witteman and colleagues’ full paper provides additional details on these and other components. The key features of existing VCMs are described in Additional file 3 Table S2 and are briefly summarized below.

#### Characteristics of studies that included VCMs

##### What was the decision context?

Types of decisions were catalogued by the decision context: whether the decision addressed 1) treatment, 2) prevention, 3) screening (other than genetic screening), or 4) genetic testing. Of the 61 VCMs, 46% focused on treatment decisions, 25% on prevention decisions, 33% on screening behaviors (not including genetic testing), and 10% on decisions about genetic testing. Three VCMs addressed two of the above decision contexts, one addressed three contexts, and one addressed all four.

##### What medium was the VCM designed for?

The VCMs reviewed were designed to be completed on paper (49%), using a computer (38%), or verbally (15%); two VCMs used two different media, thus numbers do not sum to 100%.

##### Where was the VCM located within the larger decision support tool?

Values clarification methods can be placed before or after the presentation of the relevant information needed to make a decision. The vast majority (85%) of the VCMs were presented after the information section.

##### Were decision intentions measured?

In over a third of the studies (38%), participants were not asked any questions about their decisions (intentions or actual). In 34% of the studies, participants were asked which way they were leaning, while in 28% they were asked to report their actual decision.

#### Characteristics influencing the design of the VCM

##### What theory, framework, model, or mechanism underlie the development of the VCM?

Twenty-five percent of the studies (15/61) did not report any theory, framework, model, or mechanism. Among the remaining 46 studies with any underlying structure (either apparent or reported), 39 (i.e., 64% out of the 61 total) had a theory, framework, model, or mechanism underlying the overall decision aid, which was not necessarily relevant to the VCM. Twenty-two studies out of the 46 (i.e., 36% out of the 61 total) had a theory, framework, model, or mechanism specifically underlying the VCM. It should be noted that some studies reported theory for both the VCM and the overarching decision aid; thus the numbers presented in Additional file 3 Table S2 do not sum to 100%. The most common theory was expected utility theory (18%), even though this theory makes no predictions about how VCMs can improve the process of medical decision making.

##### Was the development process described and what was the development process?

Most of the articles (74%) described, in some way, the development process of either the decision aid or the VCM. Of those that did include details, the development process used included literature reviews (42%), expert reviews (51%), and/or testing (80%). Individuals involved in the development process included health professionals (53%), academic experts (31%), and patients who have previously faced the decision (38%). Because many of the articles used multiple processes and participants in their development of the tool, the numbers in Additional file 3 Table S2 do not sum to 100%. Less than half (39%) of the VCMs in decision aids were developed and evaluated using established guidelines, most often the IPDAS standards (28% of the VCMs).

#### Characteristics of the VCM

##### What type of VCM was used?

Ten categories of VCMs were utilized. The most common types were considering the pros versus cons (46%), utility assessment with or without decision analysis (18%), prioritization (11%), and rating scales (11%) (see Additional file 3 Table S2).

##### Were the results of the VCM presented to participants?

Thirty-nine percent of the studies explicitly showed participants the result of the VCM, most of which occurred before the patient was asked to indicate their decision. Fifty-seven percent did not explicitly provide feedback to participants.

### Evaluation of the VCMs

The committee reviewed thirteen studies that compared the effects of decision support with and without VCMs and one study that compared two different approaches to using a single VCM (see Additional file 4 Table S3). The selected articles were derived from the Witteman review [8]. Only studies that included VCMs within the context of a decision aid are included here. The identified studies examined a range of health conditions, with cancer-related topics being the most common (6/13). Sample sizes ranged from small (5 of 13 studies with less than 100 participants) to moderately large (4 studies with 400 or more participants). Several different types of VCMs were employed. Available studies examined a wide range of outcomes, and no outcomes were assessed in the same manner across all or most studies. Reported outcomes included likeability of the VCM, knowledge, decision-making processes, decisional conflict, uncertainty, satisfaction, decision preference, treatment intent, actual health behaviors, regret and, in a few cases, health outcomes or cost.

The effects of the VCMs were mixed: decision processes were improved in 5 of 8 studies, but other outcomes were not measured frequently enough to reach conclusions about whether the VCMs had mainly positive or mainly neutral effects; no trials, however, suggested that VCMs led to worse outcomes (see Additional file 5 Table S4).

## Discussion: emerging issues and research areas

Although the number of studies of VCMs and decision aids is growing rapidly, our review highlights that many questions about the effects of VCMs remain unanswered. We outline several such issues here.

### Proposed theories

Above, we discussed a number of decision-making process theories without intending to establish agreement about a single theory, or a set of theories, that should be viewed as most promising in providing guidance for the design and evaluation of VCMs. More research is needed across contexts (e.g., healthcare settings) and cultures to better understand how VCMs might be designed to contribute to decision making. Such understanding requires testing VCMs based on specific theory, including theory-based predictions of anticipated effects on outcomes and consideration of how such VCMs might contribute to effective decision making.

### Intuitive processes

There is a debate currently about the value of intuitive processes in decision making. Intuitive processing is typically characterized by a lack of overt cognitive effort and the implicit integration of available information. In contrast, deliberative processing generally involves effortful, conscious and analytical thought [13]. Importantly, intuitive and deliberative processes should not be conflated with implicit versus explicit VCMs. Although explicit VCMs are often effortful, and thereby require deliberative thought, an implicit VCM may also be quite effortful and activate analytical thought processes.

From research outside of health care [14], deliberative reasoning about pros and cons may cause people to focus on attributes that are obvious, accessible, and easy to articulate, and these attributes may not be the ones that are actually the most important factors in the decision. Therefore, in contexts outside of health care, there is evidence to suggest that deliberation can cause people to ignore attributes that lead to long-term satisfaction. There is also evidence to suggest that intuitions can accurately reflect the integration of a large amount of information [13,15] However, the decisions that have been studied in the psychology literature are typically hypothetical and/or familiar decisions [cf. [16]]. There is little research yet to assess to what extent these results are expected to hold for users facing new, complex, preference-sensitive health related decisions. Until more research is available on the value of intuitive processes in such decision contexts, it is unclear to what extent a VCM that encourages intuitive processing of options would be effective to help people sort out what is most important to them. Additionally, the psychological literature described above suggests that the deliberation that is encouraged by VCMs may actually be counterproductive. Significantly more research is needed to determine whether VCMs are helpful, harmful, or neutral in terms of promoting good decision processes.

An increasing number of theories of decision making assume both intuitive and deliberative decision-making processes. Importantly, intuition and deliberation are not mutually exclusive, and extensive research shows both types of processes are used.

### How VCMs fit with patient-provider shared decision making

Researchers and practitioners need to better understand how values clarification relates to shared decision making (SDM). Is values clarification a pre-requisite to, or an element of, SDM? Does it improve SDM? Through the process of SDM, health care providers may elicit patients’ and their families’ values. Patients and their families may not necessarily be clear about their own values before the conversation with the health care team and may, on the contrary, be guided to become clear in the SDM process. Whether a VCM should precede the consultation with the health care provider, be used within the consultation, follow that conversation, or even used at all requires further study.

When VCMs are used could affect how they influence patient-provider communication. VCMs could precede the consultation, be completed during the consultation, or after the consultation. VCMs that were conducted prior to the consultation could be useful in that patients could have a better sense of their values and preferences and be better able to state their treatment preferences and goals for treatment. On the other hand, if the VCM is completed during the consultation the process itself, this could facilitate, and hopefully improve, shared decision making. VCMs conducted after the consultation may be less beneficial (especially if the patient does not see the physician following the VCM), as the patient would not have an opportunity to share potentially new information about their preferences and values.

### Use of VCMs with surrogate decision makers

More evidence is needed to determine whether VCMs would also be helpful for others involved in the decision-making processes. That is, what is the impact of VCMs designed to support the clarification of the values of those who influence the treatment decision and who are affected by the outcome of the decision (e.g., caregivers or partners of patients)? Similarly, are VCMs helpful for surrogate decision makers who are trying to make decisions on behalf of the patient and, in doing so, are trying to construct that patient’s values from their knowledge of the patient?

### Use of VCMs to reach a decision involving multiple people

More research is needed to examine how VCMs can be used to help multiple people (such as health care providers and family members) who are working together to support the patient’s decision. Specifically, little is known about how VCMs can help clarify the values that influence the advice of others to patients, as well as how VCMs could be used in a process leading to consensus about the choice (when consensus does not violate the autonomy of the patient).

### The role of distal outcomes

Attempts to develop measures of the effectiveness of VCMs have often focused on decision-making processes, likely because such processes are directly affected by the VCMs. More distal outcomes -- including effects on regret, satisfaction, behaviors, actual decisions, and measures of health -- may also be important measures of the effect of VCMs, but are often affected by many other factors. How best to incorporate these more distal outcomes into the evaluation of VCMs warrants further study.

### Implicit versus explicit VCMs

More research is needed to ascertain the "active ingredients" of a VCM, that is, the components that make independent contributions to facilitating good decision-making processes that the VCM aims to facilitate. In particular, more research is needed to clarify (a) what is required for implicit values clarification, and (b) if the use of strategies to encourage implicit values clarification is helpful, compared with explicit VCMs and also compared with no VCMs.

### How to handle more than two options

More research is needed to examine whether, in the case of multiple options, it is necessary to present all of the options, and whether multiple options should be considered simultaneously or in series. For example, it may be more helpful to identify attributes and then present only the options that match the attributes the individual who faces the decision considers most important. Or rather, it may be more helpful to present all options prior to the VCM and then identify preferred option(s) for further consideration. However, patients require sufficient knowledge of options to realize that certain attributes or values are relevant [15].

### Assessing capacity of the patient for a VCM

More research is needed to identify which types of patients are able to benefit from which VCMs, how cognitive deficits (e.g., age-related loss of executive functioning) and/or mental illness or other conditions might adversely affect the use of VCMs, and which types of VCMs are best suited for these populations.

### Empirical evidence base

Our systematic review found that the research questions and outcome variables being tested vary widely across studies. It may be helpful for studies to use at least a subset of standard measures so that study results can be more easily compared. We found that reporting of results is quite consistent, with the exception of the development process for VCMs. We recommend succinct reporting of: 1) the rationale for the design used (theory, previous designs, literature), 2) who was involved in its development (e.g., clinical experts, patients, advisory panel, etc.), and 3) how was stakeholder input incorporated (focus groups, individual interview, pilot testing, etc.). We also refer authors of reports of VCMs to Witteman et al.’s systematic review, where we report a more thorough set of categories for reporting.

## Conclusion

As described above, the theoretical and empirical basis for values clarifications research has changed significantly since the International Patient Decision Aid Standards (IPDAS) Collaboration’s 2005 review of values clarification methods in decision aids. Yet, there are still many areas that need considerable research before we can make strong conclusions about the use of VCMs in patient decision aids.

## List of abbreviations

VCM: Values clarification method; VCMs: Values clarification methods; SDM: Shared decision making; EUT: Expected utility theory

## Competing interests

Dr. Fagerlin, Dr. Kryworuchko, Dr. Scherer and Dr. Witteman have received research funding, Dr. Pignone has received research support, consulting fees, and travel support, Dr. Levin receives salary support as the research director, Dr. Reyna has received travel support and honoraria to present research at meetings and Dr. Feldman-Stewart has received travel support to teach a course on designing evidence-based decision aids from the Informed Medical Decisions Foundation, a not-for-profit (501 (c)3 ) private foundation (http://www.informedmedicaldecisions.org). The Foundation develops content for patient education programs. The Foundation has an arrangement with a for-profit company, Health Dialog, to co-produce these programs. The programs are used as part of the decision support and disease management services Health Dialog provides to consumers through health care organizations and employers.

Dr. Col has received reimbursements from private educational companies and Universities to develop and test decision aids, including Expert Medical Navigation Inc (formerly Chief Scientific Officer), EmmiSolutions LLC (Adviser), Janssen Scientific Affairs, LLC (consultant), Miami University (consultant), and University of Chicago (consultant). She has a patent pending for a computer-based decision support tool. She has received travel and/or speakers fees/honoraria from various organizations that have sponsored conferences addressing Shared Decision Making (World Congress Leadership Summit, FDA, Department of Defense) and patient engagement (Cleveland Clinic).

Dr. Pieterse was supported by a postdoctoral research fellowship from the Dutch Cancer Society.

## Authors’ contributions

AF: Conception and design, analysis and interpretation, drafting the manuscript, critically revising, final approval.

MP: Conception and design, acquisition of data, analysis and interpretation, drafting the manuscript, critically revising, final approval.

PA: Analysis and interpretation, drafting the manuscript, critically revising, final approval.

NC: Conception and design, analysis and interpretation, drafting the manuscript, critically revising, final approval.

DF: Analysis and interpretation, drafting the manuscript, critically revising, final approval.

TG: Acquisition of data, analysis and interpretation, critically revising, final approval.

JK: Conception and design, acquisition of data, analysis and interpretation, drafting the manuscript, critically revising, final approval.

CL: Conception and design, analysis and interpretation, drafting the manuscript, critically revising, final approval.

AP: Acquisition of data, analysis and interpretation, drafting the manuscript, critically revising, final approval.

VR: analysis and interpretation, drafting the manuscript, critically revising, final approval.

AS: Acquisition of data, drafting the manuscript, critically revising, final approval.

LS: Drafting the manuscript, critically revising, final approval.

CW: Conception and design, analysis and interpretation, critically revising, final approval.

HW: Conception and design, acquisition of data, analysis and interpretation, drafting the manuscript, critically revising, final approval.

## Supplementary Material

Additional file 1 **Table S1a:** Decision Process Theories. Table S1b: Examples of Decision Processes in Decision Making about Prostate Cancer Treatment Click here for file

Additional file 2 **Appendix 1:** Description of Selected Decision Processing Theories Click here for file

Additional file 3 **Table S2:** Characteristics of Values Clarification Methods and of The Studies in Which They were Presented Click here for file

Additional file 4 **Table S3:** Trials Examining Effect of a VCM vs. no VCM Within Decision Aids Click here for file

Additional file 5 **Table S4:** Outcomes Assessed in VCM Trials Click here for file

## Declarations

Publication of this supplement was partially funded by an unrestricted grant from the Informed Medical Decisions Foundation. The remaining funds were paid out of university funds (Fagerlin, Pignone). Administrative and editorial support for this supplement was also provided by The University of Texas MD Anderson Cancer Center.

This article has been published as part of *BMC Medical Informatics and Decision Making* Volume 13 Supplement 2, 2013: The International Patient Decision Aid Standards (IPDAS) Collaboration’s Quality Dimensions: Theoretical Rationales, Current Evidence, and Emerging Issues. The full contents of the supplement are available online at http://www.biomedcentral.com/bmcmedinformdecismak/supplements/13/S2.

## Endnotes

^a^ Please note that after a vigorous debate about whether the appropriate term should be patient or user, we have decided to use the term “patient” as an umbrella term for the person who is using the decision aid or participating in the VCM. We recognize that people other than patients may use the tool/VCM, but for simplicity have chosen this term.
