# Supplementary material for: Clarifying values: an updated review
Source: BMC Med Inform Decis Mak. 2013 Nov 29;13(Suppl 2):S8. doi: 10.1186/1472-6947-13-S2-S8 (PMC4044232; doi:10.1186/1472-6947-13-S2-S8)
Supplement: Additional file 1 — Table S1a: Decision Process Theories. Table S1b: Examples of Decision Processes in Decision Making about Prostate Cancer Treatment [file 1472-6947-13-S2-S8-S1.pdf]

Table S1a: Decision Process Theories

Theory	Brief Description	Identifying options	Identifying attributes of situation and/or options	Reasoning about options or attributes of options	Integrating attributes of options	Holistic comparison	Retrieve relevant values
Behavioral Decision Framework (BDF) Fischhoff, 2008 [1]	(Descriptive & Prescriptive theory): Good decision making is characterized by… (a) focusing on the consequences of options, (b) identifying all options and assessing their consequences and desirability, and (c) making trade-offs to select the alternative with the highest overall evaluation on a set of choice criteria (compensatory).	+	+		+		

Conflict Model of Decision Making (CM) Janis & Mann, 1977 [2]	(Prescriptive theory): Decisions create stress which can interfere with good decision making process, characterized by… (a) systematic information search, (b) thorough consideration of all alternatives, (c) sufficient time to evaluate each alternative, and (d) reexamine and review data in an unbiased manner			+		+	
Differentiation and Consolidation (Diff Con) Theory Svenson, 2003 [3]	(Descriptive theory): Decision making involves a process of gradually identifying differences between options (differentiation); these processes continue after the decision is made (consolidation) to minimize cognitive dissonance and future regret.	+	+	+	+	+	

Fuzzy Trace Theory (FTT) Reyna, 2008 [4]	(Descriptive & Prescriptive theory): People encode both verbatim and gist representations ("traces") of information. However, decision making is mainly determined by the gist (basic meaning for that individual) and by the social and moral values that are retrieved in context, a highly cue-dependent process.			+	+	+	+
Image Theory (IT) Beach & Mitchell, 1987 [5]	(Descriptive theory): Decision making includes two stages: (a) pre-choice screening using rapid, simple, emotionally mediated and non-compensatory strategies, followed by (b) the choice, using more deliberate and compensatory strategies with the goal to pick the choice with the most attractive expected consequences.	+	+	+	+		

Parallel Constraint Satisfaction model (PCS) Glockner & Betsch, 2008 [6]	(Descriptive theory):<br>Decision making involves<br>(a) deliberative processes for information search and production focused on actively constructing the decision problem using different decision rules for searching, editing and changing information and<br>(b) automatic processes for integrating information using an all-purpose, parallel constraint satisfaction rule.	+	+		+		
Search for Dominance Structure model (SDS) Montgomery, 2006 [7]	(Descriptive theory):<br>Decision making involves a search for a perspective that leads to optimal differences between a to-be-chosen option and other available options, in four stages:<br>(a) identifying important attributes and options,<br>(b) selecting an initially favored option,<br>(c) identifying disadvantages of the initially favored option, and<br>(d) neutralizing disadvantages of the initially favored option	+	+	+	+		

Table S1b: Examples of Decision Processes in Decision Making about Prostate Cancer Treatment

Decision Processes	Example using the clinical context of treatment of early stage prostate cancer	Theories Utilized
Identifying options	Recognizing active surveillance, surgery, and radiation as potential options	IT, SDS, Diff Con, BDF
Identifying attributes of the situation and/or the options	Identifying effectiveness of the treatments; risk of adverse effects such as impotence or incontinence; anxiety related to potential for cancer progression or metastasis	IT, SDS, Diff Con, BDF
Reasoning about attributes of options	Evaluating the potential distress of not having immediate treatment in the context of other life stresses. Recognizing the patient can change his mind about active surveillance at any time and opt for active treatment, whereas the converse is not possible.	CM, IT, SDS, Diff Con, FTT
Integrating attributes of options	Trading off fear of living with cancer vs. how important it may be to avoid adverse effects such as incontinence.	BDF, IT, SDS, Diff Con, PCS, FTT
Making holistic comparisons	Desiring to avoid surgery or radiation per se; valuing an "active" approach vs. one that feels reactive or passive	CM, Diff Con, FTT
Helping retrieve relevant values	A value that is often retrieved initially concerns survival (life is better than death) and having cancer is often equated to death. A patient retrieving only this value would likely choose immediate surgery. However, a decision aid could elicit retrieval of additional values, such as values concerning sexual health (e.g., "How important is sexual functioning to you?") that are sometimes not easily retrieved, despite their relevance.	FTT

Legend of Abbreviations
BDF: Behavioral Decision Framework
CM: Conflict Model of Decision Making
Diff Con: Differentiation and Consolidation theory
FTT: Fuzzy Trace Theory
IT: Image Theory
PCS: Parallel Constraint Satisfaction model
SDS: Search for Dominance Structure model

References

[1] Fischhoff B: **Assessing adolescent decision-making competence.** *Dev Rev* 2008, **28:**12-28.

[2] Janis I, Mann L: *Decision making: a psychological analysis of conflict, choice, and commitment.* New York, NY: The Free Press; 1977.

[3] Svenson O: **Values, affect, and processes in human decision making: a differentiation and consolidation theory perspective.** In: E*merging perspectives on judgement and decision research*. Cambridge, UK: Cambridge University Press; 2003:287-326

[4] Reyna VF: **A theory of medical decision making and health: fuzzy trace theory.** *Med Decis Making* 2008, **28:**850-865.

[5] Beach LR, Mitchell TR: **Image Theory - Principles, Goals, and Plans in Decision-Making.** *Acta Psychol* 1987, **66:**201-220.

[6] Glockner A, Betsch T: **Modeling option and strategy choices with connectionist networks: Towards an integrative model of automatic and deliberate decision making.** *Judgm Decis Mak* 2008, **3:**215-228.

[7] Montgomery H: **Decision making and action: the search for a dominance structure** In *The Construction of Preference.* Edited by Lichtenstein S, Slovic P. New York: Cambridge University Press; 2006: 342-355
