# Supplementary material for: Clarifying values: an updated review
Source: BMC Med Inform Decis Mak. 2013 Nov 29;13(Suppl 2):S8. doi: 10.1186/1472-6947-13-S2-S8 (PMC4044232; doi:10.1186/1472-6947-13-S2-S8)
Supplement: Additional file 2 — Appendix 1: Description of Selected Decision Processing Theories [file 1472-6947-13-S2-S8-S2.pdf]

Appendix 1: Description of Selected Decision Processing Theories

The *Behavioral Decision Framework (BDF)* [1-4] describes the foundations of a model of a good decision-making process (for an early precursor of this model, see Janis & Mann, 1977[5]). That is, good decision making can be characterized by the following three basic features:

1. Consequentialist decision strategies: i.e., explicitly focussing on consequences of different options/actions as opposed to non-consequentialist ones such as imitation, habit or other heuristic processing (e.g., I prefer option A because my good friend had it or my doctor seems to prefer it).
2. Thorough structuring: Identifying possible options (option generation), anticipating the consequences of options accurately (beliefs about probabilities), and determining the personal desirability of those consequences (value structuring).
3. Using compensatory decision rules: Making trade-offs using compensatory rules (e.g., trading off probabilities and consequences) rather than non-compensatory rules.

The *Conflict Model (CM)* of decision making assumes that the most thorough and ideal way of coming to a decision is by way of vigilant information processing. [5] It is characterized by (a) systematically searching information, (b) thoroughly considering all available alternatives, (c) devoting sufficient time to evaluate each alternative, and (d) reexamining and reviewing data in an unbiased manner before making a decision.

Differentiation and Consolidation (Diff Con) theory views decision making as a process over time in which one option is gradually differentiated from competing alternatives until one alternative is sufficiently superior.[6] A preliminary option is selected. The differentiation of alternatives subsequently occurs through structural differentiation, that is, changing mental representations of options in support of the preliminary choice, and process differentiation, that is, applying one or more compensatory or non-compensatory decision rules. The theory suggests that the goals of decision makers’ processing are to protect themselves against cognitive dissonance and later regret. It suggests that differentiating processes continue after the decision is made, called consolidation. That is, after a decision has been made the relative attractiveness of the chosen alternative is further upgraded to consolidate its superiority.

Fuzzy Trace Theory (FTT) proposes that people simultaneously encode mental representations (traces) of information that vary in precision. [7, 8] Verbatim traces preserve precise detail, but ‘gist’ traces preserve basic meaning and are the answer to the question “What does this information mean?” to an individual. Essential elements of a decision consist of knowledge, gist of information, retrieval (how knowledge and values are accessed when needed), and processing (how what is perceived is put together with what is retrieved to make a decision). In processing, values and principles are retrieved that are then applied to mental representations of gist. However, retrieval is highly cue-dependent (i.e., sensitive to reminders in the immediate environment), even when values and principles are strongly endorsed.[9] Main implications of FTT for values clarification would be to (a) ensure that patients understand the essential meaning of information (because different gists cue different values) (b) remind patients of an array of values, some of which might be contradictory; and (c) assist patients in applying their values to their mental representations by disentangling overlapping sets. [7, 10]

Image theory (IT) assumes two stages in making decisions: pre-choice screening of options to narrow the pool of options, followed by the choice.[11] In screening, unacceptable options are eliminated based on their incompatibility with the decision maker’s guiding principles, which underlie the adoption of goals to pursue for a specific decision. It is a rapid, simple, emotionally mediated and non-compensatory strategy that does not necessarily take place consciously. Choice of an option is accomplished by a more deliberate and compensatory strategy that evaluates both the positive and negative attributes of options. It will select the option which potentially offers the most attractive consequences. Depending on the number of options, the decision strategy will be non-compensatory followed by compensatory or compensatory alone.

The *Parallel Constraint Satisfaction (PCS) model* proposes that decision making involves deliberative processes for information search and production, and automatic processes for integrating information and making choices.[12] Processes of deliberation in decision making are mainly concerned with actively constructing the decision problem using different decision rules for search, editing and changing information regarding the decision situation. These rules are under individuals’ deliberate control, can be verbalized and give individuals the feeling that they are deciding based on reasoning. The model further assumes that individuals

integrate and structure information using an automatic all-purpose decision rule, the parallel constraint satisfaction rule.

The *Search for Dominance Structure (SDS) model* proposes that, when available options carry both advantages and disadvantages, decision making involves a search for a perspective that leads to an optimal differentiation between a to-be-chosen option and other available options -- that is, the search for dominance.[13, 14] A dominance structure is achieved when the individual perceives one option to be superior to all other options on at least one attribute and is not inferior to any other option on any attribute. The search for dominance includes four stages: selecting important attributes and options (i.e., pre-editing); finding an initially favored option for the final choice; checking disadvantages of the initially favored option (dominance testing); and neutralizing disadvantages of the initially favored option (dominance structuring).

References

[1] Fischhoff B: **Assessing adolescent decision-making competence.** *Dev Rev* 2008, **28:**12-28.

[2] Frisch D, Clemen RT: **Beyond expected utility: rethinking behavioral decision research.** *Psychol Bull* 1994, **116:**46-54.

[3] Payne JW, Bettman JR, Johnson EJ: **Behavioral Decision Research - a Constructive Processing Perspective.** *Annu Rev Psychol* 1992, **43:**87-131.

[4] Payne J, Bettman J, Luce M: **Behavioural Decision Research: An overview.** In *Measurement, Judgement and Decision making.* Edited by Birnbaum M: London Academic Press; 1998: 303-359

[5] Janis I, Mann L: *Decision making: a psychological analysis of conflict, choice, and commitment.* New York, NY: The Free Press; 1977.

[6] Svenson O: **Values, affect, and processes in human decision making: a differentiation and consolidation theory perspective.** In: E*merging perspectives on judgement and decision research*. Cambridge, UK: Cambridge University Press; 2003:287-326

[7] Reyna VF: **A theory of medical decision making and health: fuzzy trace theory.** *Med Decis Making* 2008, **28:**850-865.

[8] Kuhberger A, Tanner C: **Risky Choice Framing: Task Versions and a Comparison of Prospect Theory and Fuzzy-Trace Theory.** *J Behav Decis Mak* 2010, **23:**314-329.

[9] Bartels D, Bauman C, Skitka L, Medin D: *Psychology of Learning and Motivation. Vol. 50: Moral Judgment and Decision Making.* San Diego, CA: Academic Press; 2009.

[10] Reyna VF, Lloyd FJ: **Physician decision making and cardiac risk: effects of knowledge, risk perception, risk tolerance, and fuzzy processing.** *J Exp Psychol Appl* 2006, **12:**179-195.

[11] Beach LR, Mitchell TR: **Image Theory - Principles, Goals, and Plans in Decision-Making.** *Acta Psychol* 1987, **66:**201-220.

[12] Glockner A, Betsch T: **Modeling option and strategy choices with connectionist networks: Towards an integrative model of automatic and deliberate decision making.** *Judgm Decis Mak* 2008, **3:**215-228.

[13] Montgomery H: **Decision making and action: the search for a dominance structure** In *The Construction of Preference.* Edited by Lichtenstein S, Slovic P. New York: Cambridge University Press; 2006: 342-355

[14] Montgomery H: **Towards a perspective theory of decision making and judgment.** *Acta Psychol (Amst)* 1994, **87:**155-178.
