# Supplementary material for: Clarifying values: an updated review
Source: BMC Med Inform Decis Mak. 2013 Nov 29;13(Suppl 2):S8. doi: 10.1186/1472-6947-13-S2-S8 (PMC4044232; doi:10.1186/1472-6947-13-S2-S8)
Supplement: Additional file 3 — Table S2: Characteristics of Values Clarification Methods and of The Studies in Which They were Presented [file 1472-6947-13-S2-S8-S3.pdf]

Table S2: Characteristics of Values Clarification Methods and of The Studies in Which They were Presented

**Topic**	**N (%)**
**Characteristics of Studies in which VCMs were Presented**	
**Decision context**	**Overlapping (Note 1)**
Treatment	28 (46%)
Prevention	15 (25%)
Screening (non-genetic)	20 (33%)
Genetic testing	6 (10%)
**Medium**	**Overlapping (Note 2)**
Computer-based	23 (38%)
Online/Web	9 (15%)
CD-ROM	1 (2%)
With Multimedia	8 (13%)
Audio	3 (5%)
Video	6 (10%)
Other	2 (3%)
Decision board	1 (2%)
Paper	30 (49%)
With Audiotape	5 (8%)
With Verbal component	5 (8%)
Verbal	9 (15%)
With Visual Aids	6 (10%)
With Paper Exercises	1 (2%)
With Personal Data Assistant	1 (2%)
**Position in larger decision support tool**	
After information section	52 (85%)
Before information section	2 (3%)
Between information sections	1 (2%)
Throughout: As add-on to DA	3 (5%)
Throughout: VCE formed the entirety of the tool	3 (5%)
Unclear from article	1 (2%)
**Decision intentions**	
Asked which way leaning	21 (34%)
Asked which decision taken	17 (28%)
Not asked	23 (38%)

**Characteristics Influencing the Design of the VCM**	
**Theory, framework, model, or mechanism**	
None	15 (25%)
Underlying the VCM	
Expected utility theory	11 (18%)
Conjoint Analysis	1 (2%)
Differentiation and Consolidation Theory	2 (3%)
Multiattribute Utility Theory	2 (3%)
Other	6 (10%)
Underlying overall decision support tool	
Ottawa Decision Support Framework (ODSF)	19 (31%)
Precaution Adoption Model	3 (5%)
Edutainment Decision Aid Model (EDAM)	2 (3%)
Elaboration Likelihood Model	2 (3%)
Stages of Change	2 (3%)
Other	11 (18%)
**Development of the VCM**	
**Development process described in article**	
Yes	45 (74%)
No	16 (26%)
**Development: What aspect of development process was described**	*N.B.: Percentages out of 45 articles with descriptions*
Literature review	19 (42%)
Modification, adaptation, translation of tool	5 (11%)
Model validation	2 (4%)
Needs assessment	9 (20%)
Observation of existing processes	1 (2%)
Individual sessions, interviews	9 (20%)
Focus groups	13 (29%)
Consultations, expert review	23 (51%)
Feasibility testing	2 (4%)
Iterative process, iterative steps	9 (20%)
Prototype testing, usability testing, pilot testing	36 (80%)
**Development: Who was involved in the development process**	*N.B.: Percentages out of 45 articles with descriptions*

Clinical experts, health care professionals	24 (53%)
Experts in counseling, patient education, patient advocates	9 (20%)
Experts from relevant academic fields (e.g., epidemiology, decision-making, health communication)	14 (31%)
Plain language experts	3 (7%)
Technical experts, design experts	2 (4%)
Policymakers	1 (2%)
Consumer representatives, people from community-based groups, advocacy groups	6 (13%)
Patient experts (those who have previously faced decision)	17 (38%)
Prospective users	22 (49%)
Healthy volunteers, people recruited from community	6 (13%)
Patient advisory groups	1 (2%)
Committees, steering committees, advisory panels, multidisciplinary teams	6 (13%)
**Guidelines used**	
CREDIBLE	3 (5%)
IPDAS	17 (28%)
IPDAS cited, but not used	2 (3%)
National Health and Medical Research Council guidelines for presenting information to consumers	2 (3%)
American College of Physicians Guideline for counseling postmenopausal women about preventive hormone therapy	1 (2%)
None	37 (61%)
**Characteristics of the VCM**	
**Type of VCM**	
Decision analysis	11 (18%)
Conjoint analysis	1 (2%)
Analytic hierarchy process	1 (2%)
Tradeoffs	4 (7%)
Probability	1 (2%)
Time	1 (2%)
Attributes	2 (3%)
Pros vs. cons	28 (46%)
With weighting	23 (38%)

With binary response	4 (7%)
Viewing or listing only	1 (2%)
Prioritization	7 (11%)
Rating scales	7 (11%)
Lists of concerns	5 (8%)
List only	2 (3%)
List and discuss	3 (5%)
Social matching	1 (2%)
Other	1 (2%)
**Presentation of results**	
Yes	24 (39%)
Yes, after decision intention	3 (5%)
Yes, prior to decision intention	21 (34%)
Possibly shown explicitly (depends on options selected)	1 (2%)
No	35 (57%)
No, not at all	10 (16%)
No, not explicitly, though it may be inferred	25 (41%)
Unclear from article	2 (3%)

*This table was adapted with permission from Witteman et al.[1]

Note 1: Three VCEs address two decision contexts, one addresses three contexts, and one addresses all four.

Note 2: Two VCEs used two different media.

References

[1] Witteman H, Scherer L, Gavaruzzi T, Pieterse A, Fuhrel-Forbis A, Exe N, Kahn V, Ubel P, Feldman-Stewart D, Col N, Fagerlin A. Values Clarification Exercises: A Systematic Review. Presented at Society for Medical Decision Making Annual Meeting, Advanced Designing of Evidence-Based Patient Decision Aids, October 20, 2012. Phoenix, Arizona, USA.
