# Supplementary material for: Clarifying values: an updated review
Source: BMC Med Inform Decis Mak. 2013 Nov 29;13(Suppl 2):S8. doi: 10.1186/1472-6947-13-S2-S8 (PMC4044232; doi:10.1186/1472-6947-13-S2-S8)
Supplement: Additional file 4 — Table S3: Trials Examining Effect of a VCM vs. no VCM Within Decision Aids [file 1472-6947-13-S2-S8-S4.pdf]

Table S3: Trials Examining Effect of a VCM vs. no VCM Within Decision Aids

Reference	VCM used	Decision or context	Summary of findings relevant to VCM
Abhyankar 2011 [1] (n = 30)	Pros and Cons with weightings	Choice between standard adjuvant chemotherapy for early stage breast cancer and clinical trial testing new chemotherapy	VCM resulted in more use of personal values when evaluating attributes of options, somewhat less ambivalence, less uncertainty and did not change decision preference.
Clancy 1988 [2] (n = 1280)	Decision analysis with visual analogue scale	Choice between being immunized for Hepatitis B, screened for antibodies and immunized if negative, or not immunized unless exposed	VCM increased action-taking (screening or vaccination.)
Feldman-Stewart 2006 [3] (n = 90)	Rating (sliders)	Choice between four main options for early stage prostate cancer (watchful waiting, surgery, external beam radiation and brachytherapy)	Participants preferred VCM design with summary over VCM without summary and no VCM.
Feldman-Stewart 2012 [4]	Rating attributes	Treatment of early stage prostate cancer	VCM users reported higher preparation for decision making retrospectively and had reduced regret at 1 year
Fraenkel 2007 [5] (n = 87)	Conjoint analysis	Choice between treatments for knee pain	VCM resulted in higher scores on decisional self-efficacy, preparation for decision making, and arthritis self-efficacy

Frosch 2008 [6] (n = 611)	Time trade-off and Visual Analogue Scale	Whether or not to have prostate specific antigen (PSA) testing to screen for prostate cancer	VCM had no effect on preferences for PSA testing, preference for watchful waiting, knowledge or decisional conflict.
Kennedy 2002 [7] (n = 894)	List of concerns and discussion	Choice between treatment options for menorrhagia (advice and reassurance, addressing possible iatrogenic causes, drug therapy, or surgery such as hysterectomy or endometrial destruction)	VCM resulted in minimal improvements in self-reported health status, lower use of a more invasive treatment, higher patient satisfaction, more frequent clinician perceptions of "longer than usual" consultations, and lower overall costs.
Labrecque 2010 [8] (n = 63)	Rating scales	Whether or not to have a vasectomy	VCM had no effect on decisional conflict, knowledge, decision preferences or certainty.
Lerman 1997 [9] (n = 400)	List of concerns with discussion	Whether or not to have genetic testing for BRCA1	VCM with education resulted in increased perceptions of risks and limitations of BRCA1 testing, but knowledge was no better than education alone. Perceived personal risk decreased more with education alone, and neither VCM and education nor education alone influenced perceptions of benefits of BRCA1 testing, decision intentions, or decisions.

Montgomery 2003 [10] (n = 217)	Decision analysis with standard gamble	Whether or not to start drug therapy for hypertension	VCM increased knowledge and reduced total decisional conflict by significantly reducing scores on uninformed, unclear values and unsupported subscales and somewhat reducing scores on uncertainty subscale. VCM did not influence scores on decision quality subscale, nor did it change state anxiety, decision intention, or ultimate decision.
O'Connor 1999 [11] (n = 201)	Balance scale (Pros and Cons)	Whether or not to take hormone replacement therapy after menopause	VCM had no effect on clarity of values, concordance between values and decision, total decisional conflict, other subscales of Decisional Conflict Scale, nor acceptability of intervention.
Sheridan 2010 [12] (n = 137)	Rating and ranking tasks (prioritization)	Whether or not to initiate behaviors to prevent coronary heart disease (CHD), and, if so, which behaviors	VCM increased time spent with online tool, but did not affect decisional conflict, clarity of values, behavioral intentions, perceptions that decision was in line with values, self-efficacy for reducing coronary risk, decision intentions (including number of treatments intended), nor perceptions of tool.

van Roosmalen 2004 [13] (n = 88)	Time Trade-off	Choice between intensive screening and prophylactic surgery for breasts and/or ovaries	VCM resulted in lower scores on depression and intrusive thoughts, higher self-rated health, stronger treatment preferences for breasts, increased perceptions of having weighed pros and cons for breast treatments, and perceptions that specialists had a strong preference about breast treatments 9 months post-intervention. There were no significant differences observed for any outcomes at 3 months post-intervention.

References

[1] Abhyankar P, Bekker HL, Summers BA, Velikova G: **Why values elicitation techniques enable people to make informed decisions about cancer trial participation.** *Health Expect* 2011, **14 Suppl 1:**20-32.

[2] Clancy CM, Cebul RD, Williams SV: **Guiding individual decisions: a randomized, controlled trial of decision analysis.** *Am J Med* 1988, **84:**283-288.

[3] Feldman-Stewart D, Brennenstuhl S, Brundage MD, Roques T: **An explicit values clarification task: development and validation.** *Patient Educ Couns* 2006, **63:**350-356.

[4] Feldman-Stewart D, Tong C, Siemens R, Alibhai S, Pickles T, Robinson J, Brundage MD: **The impact of explicit values clarification exercises in a patient decision aid emerges after the decision is actually made: evidence from a randomized controlled trial.** *Med Decis Making* 2012, **32:**616-626.

[5] Fraenkel L, Rabidou N, Wittink D, Fried T: **Improving informed decision-making for patients with knee pain.** *J Rheumatol* 2007, **34:**1894-1898.

[6] Frosch DL, Bhatnagar V, Tally S, Hamori CJ, Kaplan RM: **Internet patient decision support: a randomized controlled trial comparing alternative approaches for men considering prostate cancer screening.** *Arch Intern Med* 2008, **168:**363-369.

[7] Kennedy AD, Sculpher MJ, Coulter A, Dwyer N, Rees M, Abrams KR, Horsley S, Cowley D, Kidson C, Kirwin C, Naish C, Stirrat G: **Effects of decision aids for menorrhagia on treatment choices, health outcomes, and costs: a randomized controlled trial.** *JAMA* 2002, **288:**2701-2708.

[8] Labrecque M, Paunescu C, Plesu I, Stacey D, Legare F: **Evaluation of the effect of a patient decision aid about vasectomy on the decision-making process: a randomized trial.** *Contraception* 2010, **82:**556-562.

[9] Lerman C, Biesecker B, Benkendorf JL, Kerner J, Gomez-Caminero A, Hughes C, Reed MM: **Controlled trial of pretest education approaches to enhance informed decision-making for BRCA1 gene testing.** *J Natl Cancer Inst* 1997, **89:**148-157.

[10] Montgomery AA, Fahey T, Peters TJ: **A factorial randomised controlled trial of decision analysis and an information video plus leaflet for newly diagnosed hypertensive patients.** *Br J Gen Pract* 2003, **53:**446-453.

[11] O'Connor AM, Wells GA, Tugwell P, Laupacis A, Elmslie T, Drake E: **The effects of an 'explicit' values clarification exercise in a woman's decision aid regarding postmenopausal hormone therapy.** *Health Expect* 1999, **2:**21-32.

[12] Sheridan SL, Griffith JM, Behrend L, Gizlice Z, Cai JW, Pignone MP: **Effect of Adding a Values Clarification Exercise to a Decision Aid on Heart Disease Prevention: A Randomized Trial.** *Med Decis Making* 2010, **30:**E28-E39.

[13] van Roosmalen MS, Stalmeier PF, Verhoef LC, Hoekstra-Weebers JE, Oosterwijk JC, Hoogerbrugge N, Moog U, van Daal WA: **Randomised trial of a decision aid and its timing for women being tested for a BRCA1/2 mutation.** *Br J Cancer* 2004, **90:**333-342.
