# Supplementary material for: Clarifying values: an updated review
Source: BMC Med Inform Decis Mak. 2013 Nov 29;13(Suppl 2):S8. doi: 10.1186/1472-6947-13-S2-S8 (PMC4044232; doi:10.1186/1472-6947-13-S2-S8)
Supplement: Additional file 5 — Table S4: Outcomes Assessed in VCM Trials [file 1472-6947-13-S2-S8-S5.pdf]

Table S4: Outcomes Assessed in VCM Trials

Study	Likeability	Knowledge	Decision-making Processes	Decisional Conflict	Decision Satisfaction	Regret	Preference	Intent	Behavior	Health Outcome
Abhyankar 2011 [1] (n = 30)			Improved	Improved			No effect			
Clancy 1988 [2] (n = 1280)									Increased	
Feldman-Stewart 2006 [3] (n = 90)	Improved									
Feldman-Stewart 2012 [4]			Improved			Improved				
Fraenkel 2007 [5] (n = 87)			Improved							
Frosch 2008 [6] (n = 611)		No effect		No effect			No effect			
Kennedy 2002 [7] (n = 894)					Improved				Reduced	No effect (reduced costs)

Lerman 1997 [8] (n = 400)		No effect	Improved					No effect	No effect	
Montgomery 2003 [9] (n = 217)		Improved		Improved			No effect	No effect	No effect	No effect
O'Connor 1999 [10] (n = 201)	No effect		No effect (concordance)							
Sheridan 2010 [11] (n = 137)	No effect		No effect	No effect				No effect		
van Roosmalen 2004 [12] (n = 88)			Improved				Changed			Improved

Note: After this article was drafted, Fraenkel et al. (2012) reported results of a VCM with 104 arthritis patients based on FTT that increased the proportion of patients making an informed, value-concordant choice from 35% to 64%. [13]

References

[1] Abhyankar P, Bekker HL, Summers BA, Velikova G: **Why values elicitation techniques enable people to make informed decisions about cancer trial participation.** *Health Expect* 2011, **14 Suppl 1:**20-32.

[2] Clancy CM, Cebul RD, Williams SV: **Guiding individual decisions: a randomized, controlled trial of decision analysis.** *Am J Med* 1988, **84:**283-288.

[3] Feldman-Stewart D, Brennenstuhl S, Brundage MD, Roques T: **An explicit values clarification task: development and validation.** *Patient Educ Couns* 2006, **63:**350-356.

[4] Feldman-Stewart D, Tong C, Siemens R, Alibhai S, Pickles T, Robinson J, Brundage MD: **The impact of explicit values clarification exercises in a patient decision aid emerges after the decision is actually made: evidence from a randomized controlled trial.** *Med Decis Making* 2012, **32:**616-626.

[5] Fraenkel L, Rabidou N, Wittink D, Fried T: **Improving informed decision-making for patients with knee pain.** *J Rheumatol* 2007, **34:**1894-1898.

[6] Frosch DL, Bhatnagar V, Tally S, Hamori CJ, Kaplan RM: **Internet patient decision support: a randomized controlled trial comparing alternative approaches for men considering prostate cancer screening.** *Arch Intern Med* 2008, **168:**363-369.

[7] Kennedy AD, Sculpher MJ, Coulter A, Dwyer N, Rees M, Abrams KR, Horsley S, Cowley D, Kidson C, Kirwin C, Naish C, Stirrat G: **Effects of decision aids for menorrhagia on treatment choices, health outcomes, and costs: a randomized controlled trial.** *JAMA* 2002, **288:**2701-2708.

[8] Lerman C, Biesecker B, Benkendorf JL, Kerner J, Gomez-Caminero A, Hughes C, Reed MM: **Controlled trial of pretest education approaches to enhance informed decision-making for BRCA1 gene testing.** *J Natl Cancer Inst* 1997, **89:**148-157.

[9] Montgomery AA, Fahey T, Peters TJ: **A factorial randomised controlled trial of decision analysis and an information video plus leaflet for newly diagnosed hypertensive patients.** *Br J Gen Pract* 2003, **53:**446-453.

[10] O'Connor AM, Wells GA, Tugwell P, Laupacis A, Elmslie T, Drake E: **The effects of an 'explicit' values clarification exercise in a woman's decision aid regarding postmenopausal hormone therapy.** *Health Expect* 1999, **2:**21-32.

[11] Sheridan SL, Griffith JM, Behrend L, Gizlice Z, Cai JW, Pignone MP: **Effect of Adding a Values Clarification Exercise to a Decision Aid on Heart Disease Prevention: A Randomized Trial.** *Medical Decision Making* 2010, **30:**E28-E39.

[12] van Roosmalen MS, Stalmeier PF, Verhoef LC, Hoekstra-Weebers JE, Oosterwijk JC, Hoogerbrugge N, Moog U, van Daal WA: **Randomised trial of a decision aid and its timing for women being tested for a BRCA1/2 mutation.** *Br J Cancer* 2004, **90:**333-342.

[13] Fraenkel, L., Peters, E., Charpentier, P., Olsen, B., Errante, L., Schoen, R. T. and Reyna, V. (2012), Decision tool to improve the quality of care in rheumatoid arthritis. *Arthritis Care Res*, 64: 977–985.
